# Alt Event Finder: a tool for extracting alternative splicing events from RNA-seq data

**DOI:** 10.1186/1471-2164-13-S8-S10

**Published:** 2012-12-17

**Authors:** Ao Zhou, Marcus R Breese, Yangyang Hao, Howard J Edenberg, Lang Li, Todd C Skaar, Yunlong Liu

**Affiliations:** 1 Bioinformatics Program, School of Informatics, Indiana University Purdue University Indianapolis, Indianapolis, IN 46202, USA; 2 Center for Computational Biology and Bioinformatics, Indiana University School of Medicine, Indianapolis, IN 46202, USA; 3 Center for Medical Genomics, Indiana University School of Medicine, Indianapolis, IN 46202, USA; 4 Department of Medical and Molecular Genetics, Indiana University School of Medicine, Indianapolis, IN 46202, USA; 5 Department of Biochemistry and Molecular Biology, Indiana University School of Medicine, Indianapolis, IN 46202, USA; 6 Division of Clinical Pharmacology, Department of Medicine, Indiana University School of Medicine, Indianapolis, IN 46202, USA

## Abstract

**Background:**

Alternative splicing increases proteome diversity by expressing multiple gene isoforms that often differ in function. Identifying alternative splicing events from RNA-seq experiments is important for understanding the diversity of transcripts and for investigating the regulation of splicing.

**Results:**

We developed Alt Event Finder, a tool for identifying novel splicing events by using transcript annotation derived from genome-guided construction tools, such as Cufflinks and Scripture. With a proper combination of alignment and transcript reconstruction tools, Alt Event Finder is capable of identifying novel splicing events in the human genome. We further applied Alt Event Finder on a set of RNA-seq data from rat liver tissues, and identified dozens of novel cassette exon events whose splicing patterns changed after extensive alcohol exposure.

**Conclusions:**

Alt Event Finder is capable of identifying *de novo *splicing events from data-driven transcript annotation, and is a useful tool for studying splicing regulation.

## Background

Alternative splicing is an important level of gene regulation that greatly contributes to proteome diversity [1]. It enables one gene to produce multiple isoforms that can have different biological functions. In humans, more than 90% of genes encode multiple protein isoforms [2], and many diseases are caused by the dysregulation of splicing patterns [3]. Traditionally, EST (Expressed Sequence Tags) databases and microarray technologies have been utilized to study splicing regulation [4-7]. In recent years, high-throughput RNA sequencing (RNA-seq) technology has revolutionized functional genomics by offering the most comprehensive and accurate measurements of RNAs. In addition to previously known splicing events, RNA-seq technology can be used to identify novel splicing events.

Many bioinformatics tools have been developed to derive splicing patterns from RNA-seq data. For instance, dozens of strategies have been designed for aligning RNA-seq reads. Using various strategies, such tools, including TopHat [8], MMES [9], SpliceMap [10], SplitSeek [11], G-Mo-R-Se [12], GSNAP [13] and SAW [14], enable alignment of short sequencing reads over splice junction sites even across large intronic regions. Based on such splicing-sensitive alignments, follow-up algorithms, such as Cufflinks [15] and Scripture [16] have been developed to reconstruct transcript isoforms using a genome-guided approach. Although the idea of reconstructing the whole transcriptome is intriguing, a quantitative estimate of the expression levels of each isoform is difficult, particularly for transcripts expressed at low levels and/or when more than a few isoforms exist. In addition, isoform-based approaches increase the complexity of studying splicing regulation when many isoforms are present in the sample. Event-based approaches, however, only focus on the inclusion and exclusion of individual splicing events, regardless of membership in different isoforms. This greatly reduces the computational complexity, and offers a direct path for studying splicing regulation. Based on the sequencing reads supporting inclusion and exclusion events, MISO (mixture of isoforms) [17] is designed to estimate the percentage of inclusion for every previously documented alternative-splicing event in a sample. It further offers a probabilistic framework for detecting differentially regulated exons, and provides functional insights into pre-mRNA processing.

One requirement for implementing MISO is to provide a pre-defined alternative event annotation. Such an annotation heavily relies on previous knowledge, and is not complete or even available for many species. For instance, in the official MISO release, alternative splicing annotation library [17] is only available for human, mouse, and Drosophila genomes, and does not allow event-based analysis on datasets from other species. In addition, even for the species whose alternative splicing has been heavily investigated, identifying novel splicing events can be important. Therefore, having a tool for detecting novel splicing events directly from RNA-seq data is desirable.

In this study, we developed a tool, *Alt Event Finder*, for generating *de novo *annotation for alternative splicing events from a map of transcripts and isoforms reconstructed from RNA-seq experiments. In conjunction with upstream alignment and isoform reconstruction tools, we demonstrated that *Alt Event Finder *has the ability to identify novel cassette exon events that are not documented in the established databases. We evaluated the performance of this strategy with different combinations of alignment and transcript reconstruction algorithms, using a human dataset where alternative splicing events have been extensively investigated. We further implemented this tool on an RNA-seq dataset from rat genome, for which alternative-splicing annotation is not available.

## Results

### Workflow

**Figure 1:**
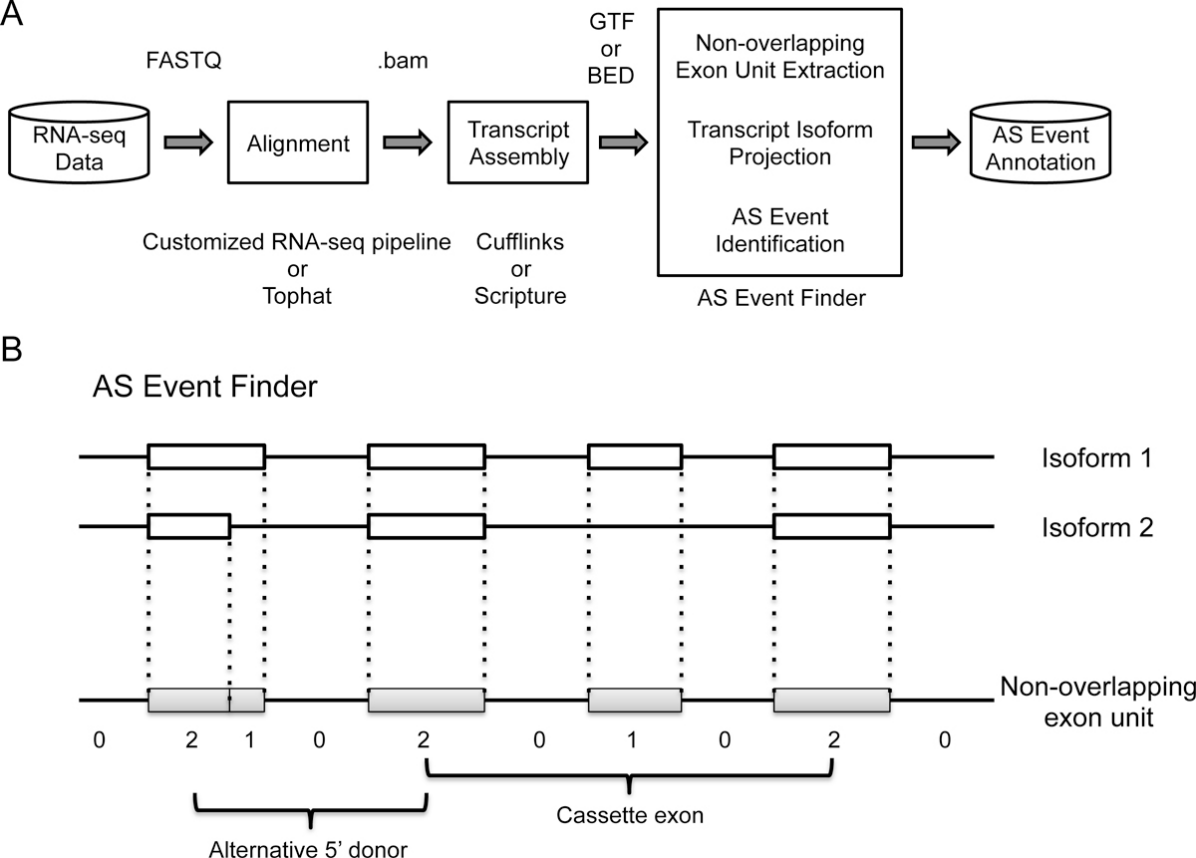
**Workflow of the alternative splicing event identification pipeline**. A) RNA-seq-derived transcriptome data was aligned using a customized RNA-seq pipeline based on known splicing junctions or Tophat. Cufflinks or Scripture was used for isoform annotation. Based on the data-derived transcript annotation, *Alt Event Finder *was applied to identify the novel alternative events. B) Strategies of Alt Event Finder for de novo event detection.

As shown in Figure 1A, the input to *Alt Event Finder *is a mixture of RNA isoforms identified from transcriptome reconstruction tools, such as Cufflinks [15] or Scripture [16]. The output is a list of alternative splicing events directly derived from isoform annotation. *Alt Event Finder *includes two major steps. First, based on a GTF or BED file for isoform annotation, the unions of the exon regions are split into the smallest units that do not overlap with each other in the genome space, or minimum non-overlapping exon units. This design is similar to the PSR (probe selection regions) definition for Affymetrix exon arrays [18], and can reflect the complexity of the exon structures where alternatively spliced exons from the same gene may overlap (i.e. alternative donor or acceptor sites). Second, individual transcript isoforms (identified from transcriptome reconstruction tools such as Cufflinks and Scripture) will be projected to the non-overlapping exon units (Figure 1B). The number of isoforms containing each unit is recorded. Special strings of such numeric patterns will be used for deriving different types of splicing events. For instance, for a gene with two isoforms, a string of [2-0-1-0-2] indicates the presence of a cassette exon. Although this report focuses on cassette exons, such simple design allows extension to other types of events easily.

### Alternative splicing event annotations from human liver data

To test the performance of our strategy, we implemented *Alt Event Finder *on a RNA-seq dataset derived from human primary hepatocytes; the RNA-seq experiment was conducted using the SOLiD 5500×l system (Life Technologies). The dataset consists of 7 pairs of samples derived from 7 individuals. Each pair includes a drug exposed sample and a control sample. To test the performance of *Alt Event Finder *on data with various sequencing depths, in addition to the 14 RNA-seq samples, we created 7 patient-specific datasets by merging the exposed and control samples from the same individual; and 1 hepatocyte-specific dataset by merging all the 14 samples together.

**Figure 2:**
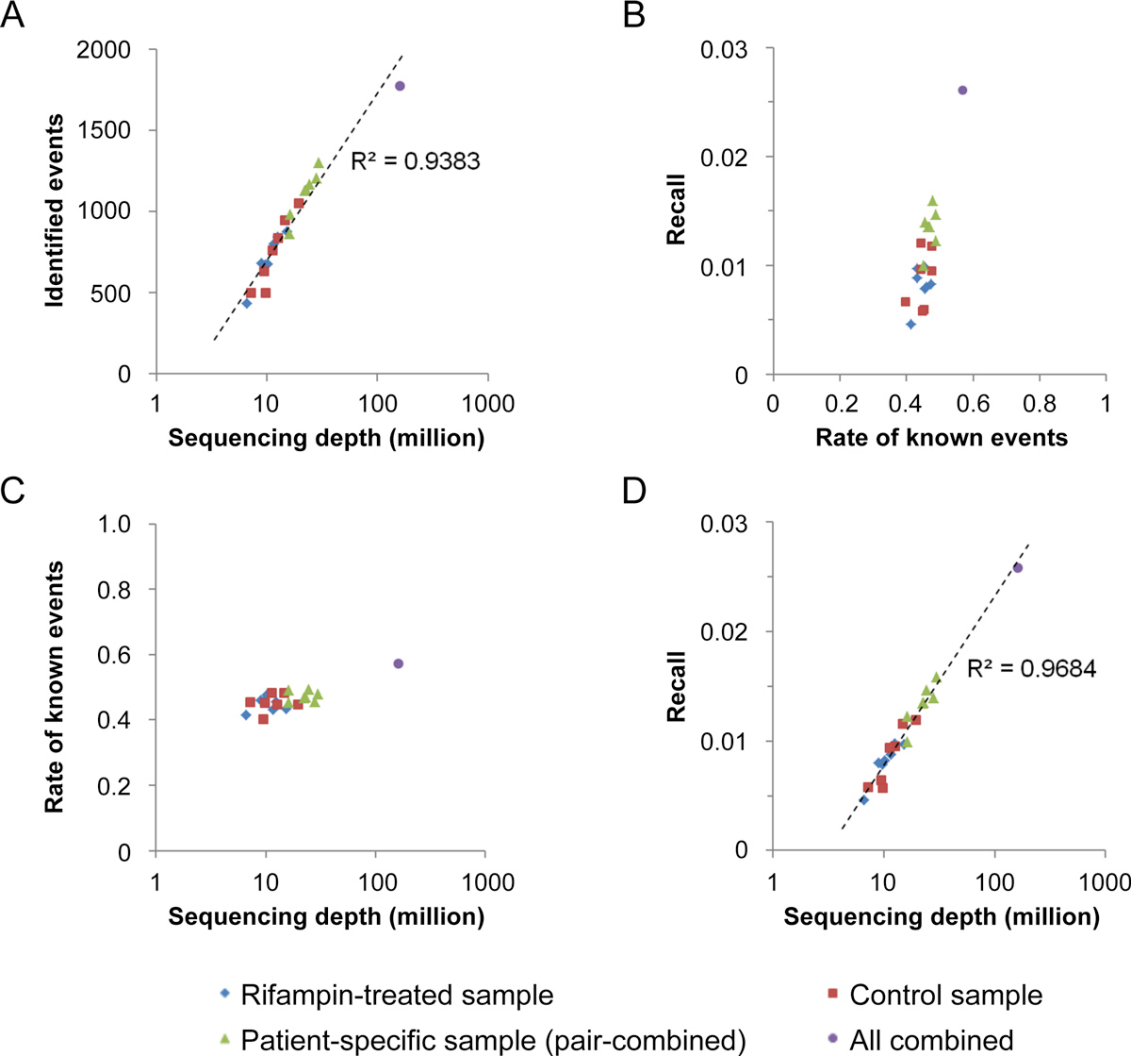
**Performance assessment for the Alt Event Finder**. A) The total number of identified events increases with sequencing depth. Each dot corresponds to a sample; the color and the shape of the sample denote its biological condition; the regression line is displayed in dashed line with an R-squared value of 0.9383; B) Performance for the *Alt Event Finder *pairing with a customized alignment pipeline and Cufflinks. The X-axis (rate of known events) is defined by the number of overlapping events divided by the number of data-driven events, and Y-axis (recall) is defined as the number of overlapping events divided by the number of events in the official MISO annotation library; C) The rate of known events does not change with sequencing depth; D) The recall rate of the *Alt Event Finder *increase linearly with the logarithmic transformation of the total mappable reads. The regression line is displayed in dashed line with an R-squared value of 0.9684.

We used BFAST [19] as the primary aligner of short reads, due to its higher sensitivity on color-space data [20]. The alignment was conducted on both genomic DNA sequences and a junction library including all the combinations of known junction boundaries (within a 100 kb span) annotated in the UCSC Gene database. The total number of mappable reads in each sample ranged from 6.6 million to 19.5 million. We then used Cufflinks [15] to reconstruct transcript isoforms. From that, we applied *Alt Event Finder*. The number of identified alternative splicing events increased as a function of depth of coverage (Figure 2A); events ranged from 433 to 1,049 in individual samples, from 761 to 1,298 for patient-specific datasets (combining control and treated data), and was 1,771 for all the samples combined.

To evaluate the performance of the proposed strategy, we compared our data-derived events with known alternative splicing events documented in the MISO release (based on UCSC hg19 assembly) [17]. For each sample, we calculated a rate of known events (RKE), which measures the percentage of identified events that were in the known splicing events annotation, and a recall value, which was calculated as the percentage of known splicing events that were recovered by our strategy. As shown in Figure 2B, the rate of known events varies from 0.4 to 0.57. This indicates that a significant portion of splicing events we detected was not documented in the current database, although junction reads were found in support of their existence. The recall values, however, are low, ranging from 0.004 to 0.025. This is not surprising since the known event annotation aims at completeness, and therefore documents events from many tissues with a variety of biological conditions; most of these events should not be present in one tissue under one or two biological conditions. We further evaluated the relationship between sequence depth and rate of known events (Figure 2C) and racall values (Figure 2D). Rate of known events do not show apparent changes, suggesting that the genes expressed at lower levels contain a similar percentage of novel events as the more abundant transcripts, but they require greater sequencing depth to identify. The recall, however, increases almost linearly with logarithmic transformation of the total number of mappable reads. These results (Figure 2C and 2D) indicate that many more events will be identified with deeper sequenced samples, while the percentage of novel events doesn't change. Therefore, more novel events will be identified from deeper sequenced data.

**Figure 3:**
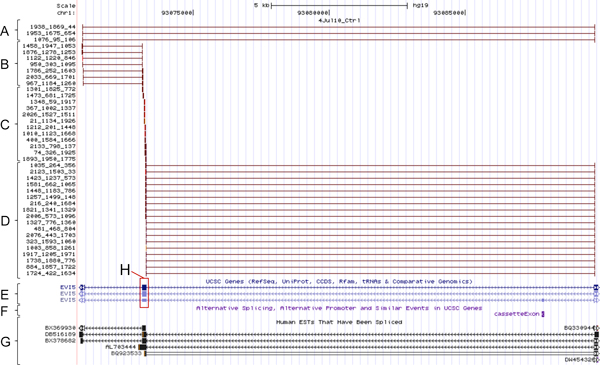
**Screenshot of one of the novel cassette exon events**. The diagram demonstrates reads supporting a cassette exon event that was not previously documented. This gene locates on the reverse strand. The genomic loci of the cassette exon, the 5' and 3' constitutive exon are chr1: 93073138-93073284, chr1: 93089733-93089891, and chr1: 93070864-93070959, respectively. A) Sequencing reads supporting the junction of the 5' and 3' constitutive exons, which indicates exon exclusion; B) Sequencing reads supporting the junction of the cassette exon and the 3' constitutive exon; C) Sequencing reads supporting the transcription of the cassette exon; D) Sequencing reads supporting the junction of the 5' constitutive exon and the cassette exon; E) UCSC gene annotation track; F) Alternative splicing annotation track; G) Human EST track; H) The cassette exon in UCSC gene annotations. No previous evidence of this cassette exon event was shown in the gene annotations, alternative splicing event annotations or the EST records.

Figure 3 is the screen shot of one of the novel cassette exons (chr1: 93073138-93073284) not documented in either the official MISO annotation library (based on UCSC hg19 assembly) [17] or the *Alt Event *track (Figure 3F) in the UCSC Genome Browser (GRCh37/hg19, Feb. 2009) [21]. As shown in the figure, 40 reads are identified around this exon (Figure 3ABCD), of which 37 (Figure 3BCD) support inclusion events (exonic reads on the alternative exon, and junction reads connecting the upstream or downstream exon with the alternative exon), and 3 (Figure 3A) support exclusion events (reads connecting upstream and downstream exons directly), respectively. Importantly, the presence of 28 exclusive junction reads provides a strong evidence for the presence of this novel event.

**Figure 4:**
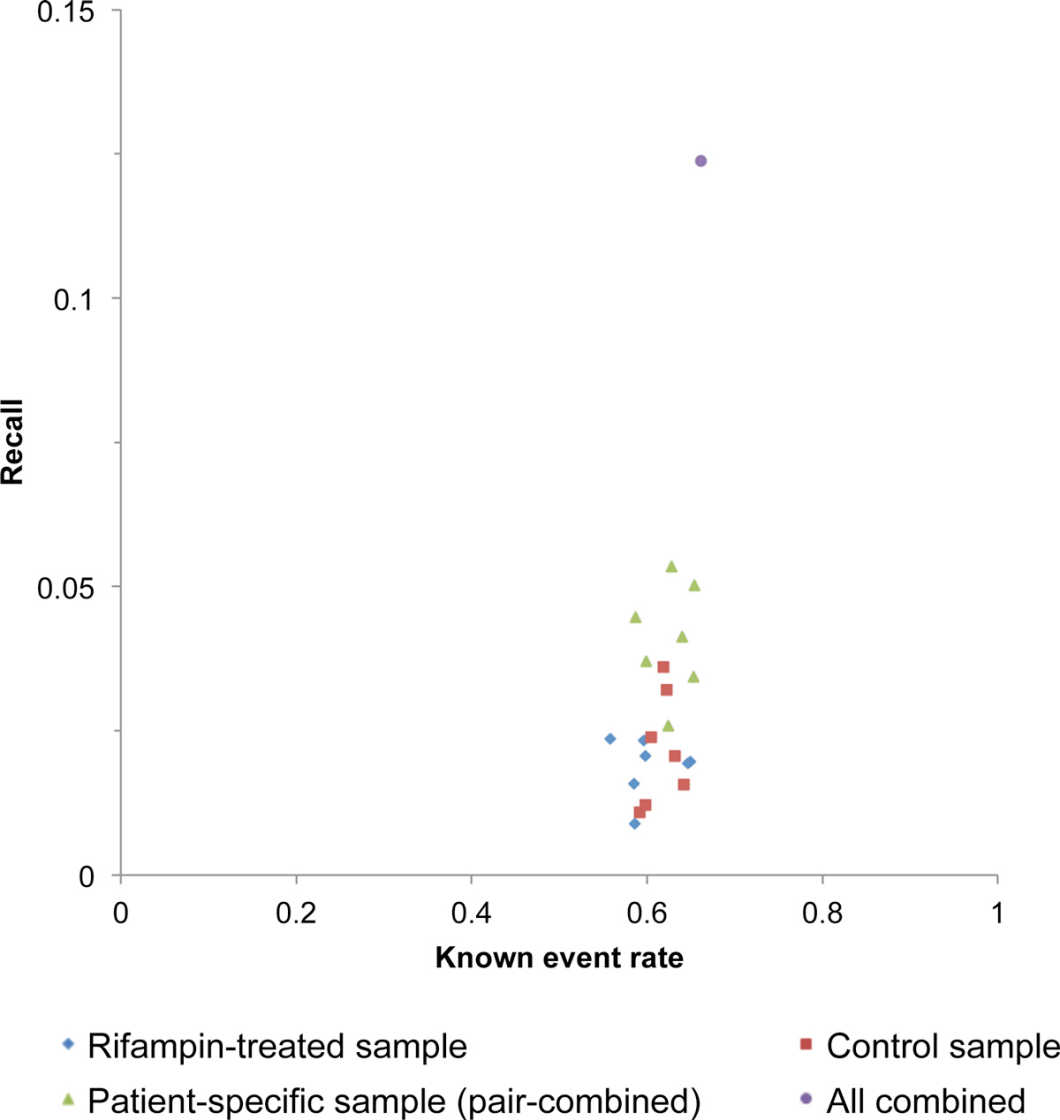
**Performance with adjusted known event annotation**. The rate of known events and recall values were calculated only based on the events that have at least 10 junction reads supporting the inclusive event and 1 junction read supporting the exclusive event.

Due to the tissue specific nature of gene expression and alternative splicing, using all the known events in the human genome cannot fairly evaluate the sensitivity of the proposed approach. This is either due to the absence of certain isoforms in a specific tissue, hepatocytes in this case, or because the overall gene expression levels are too low to be detected given a specific sequencing coverage. We therefore removed the events either with low expression levels, or with extremely unbalanced inclusion/exclusion ratio, from the overall alt event library. For the latter, it is possible that the RNA-seq data can only detect the isoforms with inclusion or exclusion events, but not both. To fairly evaluate the performance, we derived exon inclusion and exclusion ratios using MISO, based on all the annotated splicing events. We further filtered the annotated events containing no less than 10 reads supporting inclusion and 1 read supporting exclusion. After applying this filtering, for the hepatocyte-specific sample (merging reads from all the 14 samples), 83.4% of the 39,232 total annotated cassette exon events were removed. The adjusted recall rate is shown in Figure 4. Clearly, for individual samples, the recall remains low, ranging from 0.9% to 3.6%. This number increased for patient-specific samples (merging control and drug treatment for one individual), ranges from 2.6% to 5.0%, and 12.4% for all the 14 samples combined. This low recall rate may be due to the stringent threshold of Cufflinks, which aims at maximizing specificity.

### Selection of alignment and transcriptome reconstruction tools

We further evaluated how the performance of the *Alt Event Finder *is influenced by the alignment and transcriptome reconstruction tools. For the alignment tool, in addition to our customized RNA-seq pipeline which focus on known splicing junctions, we also tested TopHat [8], one of the most widely used RNA-seq alignment software. For the transcriptome reconstruction tool, in addition to Cufflinks [15], which aims at maximizing specificity, we have also tested Scripture [16], a computational algorithm aiming at higher sensitivity.

**Figure 5:**
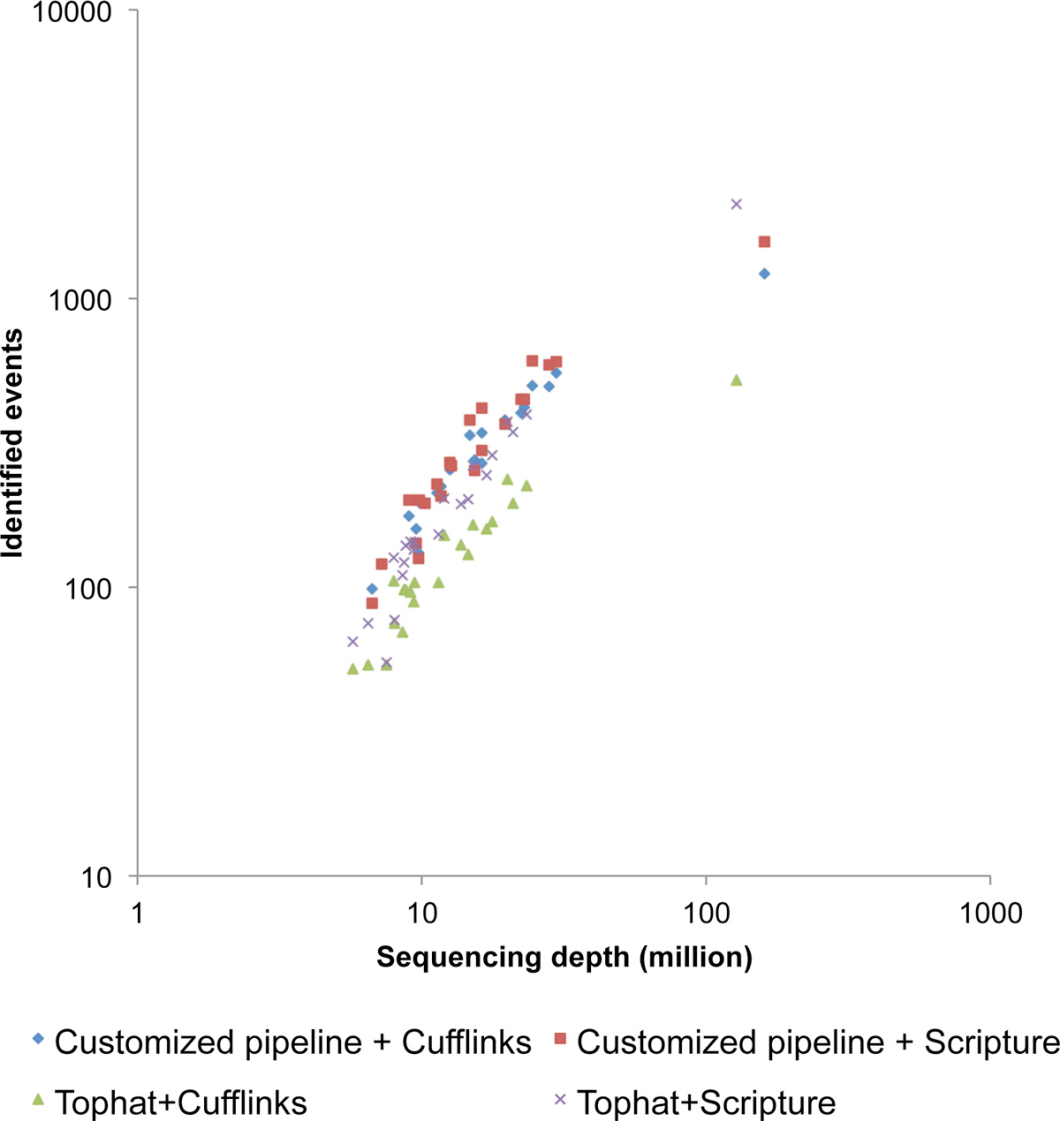
**The number of identified events differs with different combinations of alignment and transcript reconstruction algorithms**. Each dot describes a sample. X and Y axes denotes sequencing depth and the total number of identified events. Samples were color-coded based on their combinations of upstream algorithms.

The total number of events identified based on 4 different strategies (Customized RNA-seq pipeline and Cufflinks, Customized RNA-seq pipeline and Scripture, Tophat and Cufflinks, and Tophat and Scripture) varies significantly (Figure 5). At low sequencing coverage, the customized RNA-seq pipeline (using BFAST and annotated exon boundaries) consistently identified more events. When the sequencing depth is higher than 100 million reads, however, our AS identification pipeline offers significantly more events when Tophat is partnering with Scripture (Figure 5). When comparing two transcriptome reconstruction tools, Scripture offers higher number of events regardless of the sequencing depth and sequencing alignment algorithm (Figure 5). Among all the four strategies, the combination of Tophat and Scripture at high sequencing coverage identified highest number of events.

### Identify alternative splicing events in the rat genome

We applied *Alt Event Finder *to study the alcohol-induced alternative splicing changes in liver tissue, using alcohol-preferring rats as a model system. Seven female rats were heavily exposed to alcohol for 10 weeks followed by 2 weeks without alcohol, and another 7 were not subjected to alcohol exposure (controls). An RNA-seq experiment was conducted on the liver tissues. After sequence alignment using TopHat, 123,017,701 and 92,389,972 total reads were mapped in the 7 control and 7 alcohol-exposed animals, respectively. Scripture was used for transcript reconstruction. Alt Event Finder identified 505 candidate events with a mixture of multiple isoforms in the combined sample of all 14 rats. With a MISO isoform differential expression test, we found 75 were alternatively spliced at Bayesian Factor (BF) [17] larger than 2; this number implies that it is twice as likely for the events to be alternatively spliced than not. A more stringent cutoff derived 55 events with BF > 5.

**Figure 6:**
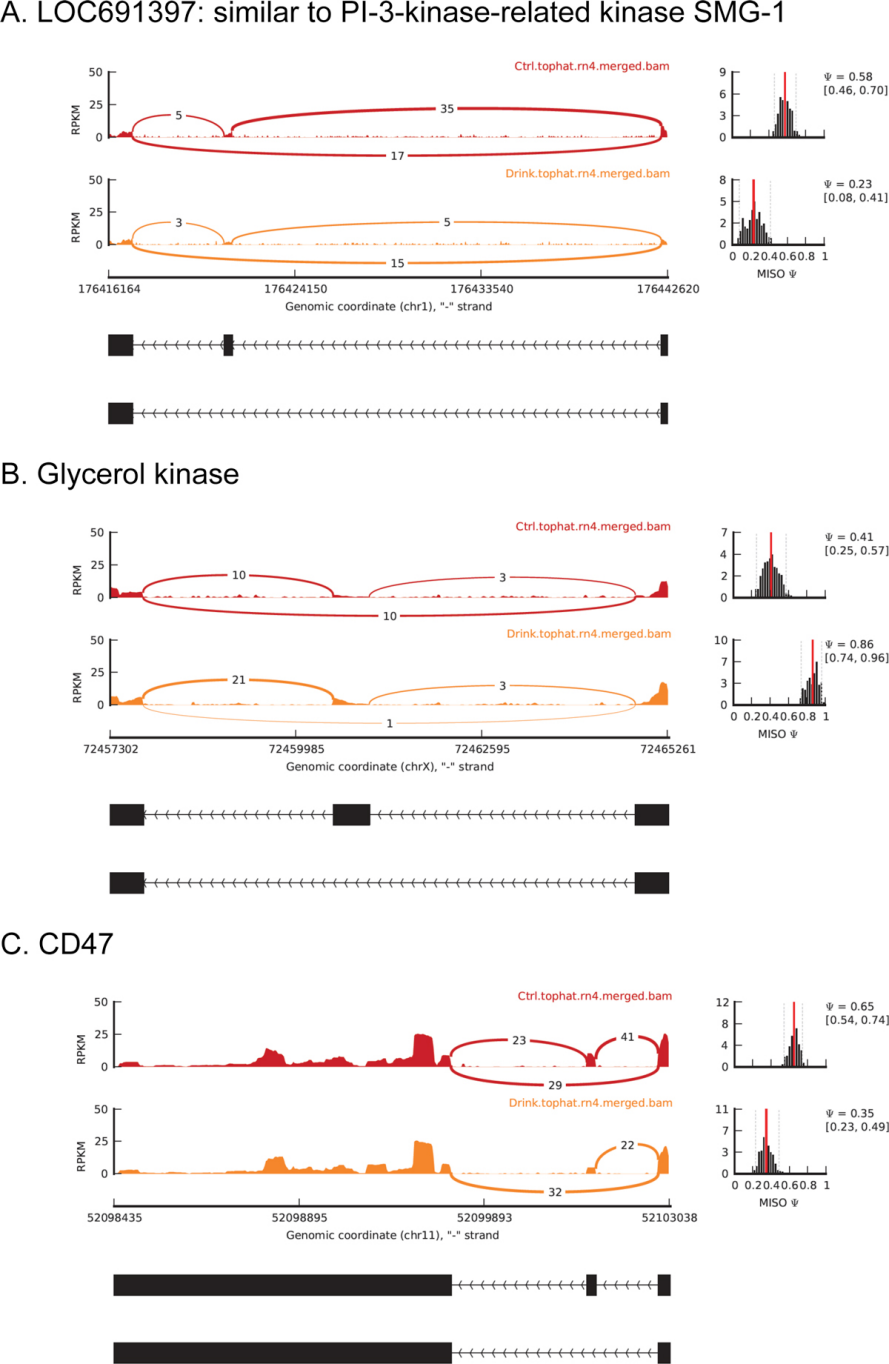
**Sashimi plot for three novel events that are alternatively spliced in rat liver with chronic alcohol exposure**. The RNA-seq read densities supporting inclusion and exclusion events are shown in the figure. The estimated percentage of inclusion for the alternative events and their estimated confidence intervals are also demonstrated. The Sashimi plot is produced by the MISO package.

Figure 6 shows the Sashimi plots [17] for three events with apparent alcohol-induced splicing changes in genes highly expressed in liver tissues, LOC691397 (similar to PI-3-kinase-related kinase SMG-1), Glycerol kinase, and CD47 (Figure 6A). For LOC691397, 40 junction-reads support exon inclusion in the control samples, and 17 support exclusion. In the alcohol exposed samples, however, these numbers changed to 8 and 15, respectively. This pattern indicates that chronic alcohol exposure induces higher relative expression levels of the isoforms without the cassette exon, with a BF value 15.37. Similarly, CD47 showed lower inclusion ratio after alcohol exposure (Figure 6C), while alcohol drinking induces exon inclusion for the glycerol kinase (Figure 6B).

## Discussion

In this study, we developed a tool, *Alt Event Finder*, which generates splicing event annotations from RNA-seq data. Most event-based analysis, such as MISO [17], cannot work without a library of known event annotations. Therefore they cannot be implemented on a genome for which annotation is unavailable, such as the rat genome. Even for a genome for which alternative splicing has been extensively studied, such as human or mouse, lack of a *de novo *event finding tool limits the power of studying events that are not previously documented. Alt Event Finder bridges the gap between event-based analysis and isoform-based transcriptome reconstruction algorithms, such as Cufflinks and Scripture. It's an important addition to the current AS analysis toolset.

Our algorithm extracts "minimum non-overlapping exon units" (Figure 1B) from RNA-seq-derived transcript isoform annotation based on Cufflinks or Scripture, and further identifies potential alternative events. This strategy greatly increases the flexibility of our methods. Although the current study focuses on cassette exon, it can be easily extended for other types splicing events, such as intron retention, alternative 5' donor, alternative 3' acceptor, and so on. This is important because certain types of events can be more prevalent in specific tissue types. For instance, cassette exons are dominant in brain tissues, while alternative 5' donor and 3' acceptor events are more abundant in liver tissues [22].

*Alt Event Finder *relies on upstream alignment and isoform reconstruction tools. We have evaluated how different tool combinations affect the ability to discover novel splicing events. We found that a customized alignment pipeline based on known exon boundaries perform better in low sequencing coverage (< 100 Million reads), while TopHat did better for high sequencing coverage. This is because TopHat derives exon structures mainly based on the accumulation of RNA sequencing reads. Since it does not rely on existing exon annotations, at lower coverage, the data may not have adequate power to properly identify low expressed exons. For higher coverage, however, TopHat will not only have enough power to precisely map the boundaries of known exons, but also be more suitable for identifying novel exons. We have also found that we can generally identify more AS events using Scripture as isoform reconstruction tool, compared to using Cufflinks, because Scripture aims at maximizing sensitivity, while Cufflinks aims at specificity. Overall, we recommend using the mapping algorithm based on known exon annotation and Scripture combination at a low sequencing depth, and the TopHat and Scripture strategy with high sequencing coverage.

To find out the cause of the low recall rate, we investigated the AS events that were identified with the official MISO library but not found in our annotations. One of the major causes of such events is lack of junction reads between the cassette exon and constitutive exons, which makes the inclusive isoform not detectable by Cufflinks and Scripture, but still quantifiable by MISO since reads are covering the cassette exon. Another cause is additional alternative spliced 3' and 5' sites on a cassette exon event, which make an event in our annotation different from the official MISO annotation.

Since *Alt Event Finder *is a data-driven approach, its power highly depends on the sequencing depth. When the sequencing depth is low, a lot of junction read will be missed, and a lot of low expressed exons could be "disconnected"; this will significantly decrease the power of the transcriptome reconstruction algorithm for rebuilding the isoforms from RNA-seq data, therefore affect the performance of *Alt Event Finder*. Therefore, when possible, increasing the sequencing depth can significantly elevate the power of novel event identification.

When deep sequencing data is not available, at the *de novo *event identification step, we recommend pooling sequencing reads from all the samples. This will enable identification of the events that lowly expressed in individual samples. It will also enable us to identify the events that have complete inclusion in one condition, but exclusion in another. These events cannot be identified within individual samples, but the inclusion/exclusion switches are enormously interesting.

## Methods

### Dataset

#### RNA-seq dataset

We used two RNA-seq datasets for *de novo *alternative splicing event identification, human hepatocytes and rat liver cells. In the human study, primary hepatocytes were isolated from seven individual subjects, and treated with Rifampin. Total RNA from both control and treated samples were extracted. RNA-seq experiments were conducted using the SOLiD 5500×l system with the standard protocol. In the rat study, RNA-seq experiments were conducted on liver tissues from 7 non-drinking alcohol-preferring rats, and 7 alcohol-preferring rats that were heavily exposed to alcohol for 10 weeks followed by 2 weeks without alcohol. The experiment was conducted on the SOLiD 4 system with the standard protocol.

#### Known splicing event annotation

The known alternative splicing event annotation for human genome was retrieved from the official MISO library (based on UCSC hg19 assembly). The annotation file was generated based on transcript annotation using an EST database; a splicing event was considered alternative if it was supported by multiple ESTs.

### RNA-seq alignment

We used two RNA-seq alignment pipelines, TopHat [8] and a customized strategy using BFAST [19] as primary aligner and known splicing sites documented in UCSC Known Gene database [23]. TopHat v1.4.0 was used with standard parameter settings on color space data. The customized pipeline uses BFAST [19] as a primary aligner due to its computability with small insertions/deletions, and reported higher sensitivity on color space data [20]. The overall alignment of our customized RNA-seq pipeline includes two levels, alignment on genomic DNA sequences, and alignment on a junction library based on all possible exon combinations within a 100,000-bp span, based on documented exon boundaries. This is different from TopHat strategy, which uses sequencing reads enrichment and splicing sequence features (GU...AG) for exon boundary detection.

### Other algorithms for splicing analysis

Based on the alignment output from TopHat or the customized pipeline, Cufflinks v1.2.1 [15] and Scripture [16] were used for isoform reconstruction. fastMISO (Mixture of Isoforms) [17] was used to calculate the percentage of inclusion for annotated and novel alternative splicing events. Standard parameter settings were used for all the three programs.

### *De novo *alternative splicing event identification

As shown in Figure 1A, Alt Event Finder uses transcript isoform annotation from Cufflinks (GTF format) or Scripture (BED format) as input. The output is the data-derived alternative event annotation in GFF3 format, which can be used as MISO input. From the isoform annotation, the Alt Event Finder extracts "minimum non-overlapping exon regions" as expression units (Figure 1B), counts the number of isoforms that include each expression unit, and further derives appropriate AS events based on the string of counts (Figure 1B). In this study, we focus on cassette exons.

### Performance assessment

The ability of *Alt Event Finder *was evaluated by comparing with the splicing event annotation in the MISO library. Events from two annotations are considered consistent only if the genomic loci of the alternative exon (cassette exon) and their 5' upstream and 3' downstream exons are identical. This ensures the most conservative evaluation. The performance of *Alt Event Finder *is assessed by using three measurements, the total number of identified events, and the rate of known events and the recall of the overall finding. The rate of known events is defined by the percentage of known events within data-driven ones, and recall is defined as the percentage of data-driven events within known ones.

## Competing interests

The authors declare that they have no competing interests.

## Authors' contributions

AZ and YL designed the study, conducted the analysis, and drafted the manuscript. MB and YL designed the customized RNA-seq alignment pipeline. YH assists the event-based analysis. HJE provided RNA-seq data on rat liver and suggestions on analyses and interpretation, LL and TCS provided RNA-seq data on human hepatocytes. All the authors read and approved the final manuscript.
